# Armed conflict: the “perfect storm” for One Health

**DOI:** 10.3389/ijph.2026.1610237

**Published:** 2026-09-04

**Authors:** Oliver Razum, Jonas Rickermann, Andrea Sylvia Winkler

**Affiliations:** 1 Department of Epidemiology and International Public Health, School of Public Health, Bielefeld University, Bielefeld, Germany; 2 Sydney Health Ethics, School of Public Health, The University of Sydney, Sydney, NSW, Australia; 3 Department of Neurology, Technical University of Munich University Hospital, and Center for Global Health, School of Medicine and Health, Technical University of Munich, Munich, Germany; 4 Department of Community Medicine and Global Health, Institute of Health and Society, Faculty of Medicine, University of Oslo, Oslo, Norway

**Keywords:** armed conflict, communicable diseases, environment, One Health, zoonoses

Recent outbreaks of Hantavirus on a cruise ship and of Ebola in the Democratic Republic of the Congo have increased public interest in the One Health concept and its implementation. These outbreaks are linked to One Health through the fundamental idea that the health of humans, animals, and ecosystems (including plants, soil, water, and air) is interconnected and interdependent, and therefore must be considered together to protect and improve species and ecosystem health overall. According to the One Health High-Level Expert Panel (OHHLEP), this interdisciplinary approach combats health risks by focusing on clean water, energy, air, safe and nutritious food, climate, and sustainable development [1].

Additionally, the Lancet One Health Commission (LOHC) has compiled a substantial body of evidence on One Health [2], and most recently, high-level political actors have reaffirmed their commitment to advancing One Health through the Lyon One Health Commitments [3]. These documents elaborate on much of the current international scientific and policy agenda for One Health but fail – similar to so many other One Health publications – to consider a key geopolitical phenomenon with a significant impact on health: armed conflict. Wars in Sudan, Ukraine, and Gaza have made it clear that armed conflicts are a “perfect storm” for One Health. By “perfect storm” we mean that several harmful mechanisms occur simultaneously, reinforce one another, and produce greater damage together than they would individually. We will show why armed conflict fulfills all three elements of this definition.

The first feature of this “perfect storm” is its simultaneous impact on all three domains of One Health. Armed conflict destroys ecosystems, including agricultural land, and alters animal habitats. It displaces people [4] and domestic and wild animals. At the same time, it weakens the institutions needed for a response, particularly those related to governance, health, and surveillance. Together, these processes create cascading effects across human, animal, and environmental health that exceed the impact of each process in isolation.

The “storm” unfolds through direct and indirect pathways, affecting each domain of One Health. For example, armed conflicts destroy ecosystems, resulting in negative consequences for water, air, and soil [5]. The burning oil fields in Tehran following bombing attacks illustrate these effects [6]. The toxic smoke from these conflict-related destructions can lead to respiratory diseases and an increased risk of cardiovascular complications in humans [6]. Animal health is similarly affected, particularly in aquatic ecosystems exposed to emissions and black rain [6].

Another component of this perfect storm is the pollution of water and food, and the destruction of critical infrastructure, such as healthcare facilities [7]. Damaged sewage systems become breeding grounds for vectors such as mosquitoes and flies, along with rodents, increasing infection risk and the potential for spillover of pathogens [7]. Furthermore, conditions in conflict zones promote the emergence and spread of antimicrobial resistance (AMR) [8]. In Ukraine, for example, AMR is driven by the provision of mass trauma care to patients with complex wounds, their long-distance transfer, and the improper use of antibiotics [9].

It is striking that armed conflict remains largely neglected within One Health, whether in official statements such as the Lyon Commitments (except for President Macron’s warning that war is among the forces undermining international cooperation) [3], the OHHLEP definition [1], or the LOHC [2]. While armed conflict creates interacting risks across the One Health spectrum, research often analyzes only its individual components. Scientific studies frequently focus on spillover events and their effects on human health, whereas studies of the environmental impacts of armed conflicts often fail to consider their consequences on human or animal health. This fragmentation probably has historical roots: One Health developed around zoonoses. Later, climate change, pollution, and biodiversity loss became major concerns, while armed conflict continued to be viewed as an external geopolitical issue rather than a determinant of One Health.

Failing to recognize this “perfect storm” has practical consequences. If armed conflict is already absent from the conceptual framework of One Health, then it is likely also to be absent from funding priorities, surveillance systems, preparedness plans, rebuilding strategies, governance structures, and educational curricula. Recognizing armed conflict as the archetypal “perfect storm” for One Health would not merely add another topic to the agenda. It would fundamentally broaden the scope of One Health, shifting the focus from responding to biological and environmental threats to addressing one of their most powerful upstream drivers. One Health strategies, research, and governance need to consider and prioritize the detrimental effects of armed conflicts [10, 11]. Preparing for and mitigating this “perfect storm” requires documenting conflict-related harms, building back better in line with One Health principles, preventing war, and fostering peace and political stability.
